# OSAS in children: Where are we?

**DOI:** 10.1590/S1808-86942011000300001

**Published:** 2015-10-19

**Authors:** Leila Azevedo de Almeida, Wilma Terezinha Anselmo-Lima, Fabiana Cardoso Pereira Valera

**Affiliations:** Assistant Physician - Neurology - HCFMRP-USP; Associate Professor of Otorhinolaryngology - FMRP-USP; PhD; Professor of Otorhinolaryngology - FMRP-USP

## SAOS na infância: Onde estamos?

In recent years, thanks to obvious developments in Sleep Medicine, we have had a better definition of the pathophysiological, clinical and therapeutical aspects associated with high prevalence disorders, such as the Obstructive Sleep Apnea Syndrome (OSAS). While the parameters are well defined and the outcomes have been better studied in the adult population; as far as children are concerned, medical literature is scarce in this context, which nonetheless has not prevented the development of new paradigms associated with Sleep Apnea in Children.

It is widely known than OSAS cause facial morphometric changes, behavioral and cognitive disorders, as well as hyperactivity and attention deficit. Nonetheless, more recently it has been reported that it is also associated with metabolic and cardiovascular disorders, as well as an increase in insulin resistance and high blood pressure, amongst others, and it also causes the development of OSAS in adulthood. Having these new repercussions, childhood OSAS must be diagnosed and dealt with aggressively.

In the Second Edition of the International Classification of Sleep Disorders, from 2005, childhood OSAS is defined based on clinical and polysomnographic criteria: complaint of snoring and difficult breathing during sleep, associated with other OSAS signs and symptoms, and polysomnography tests showing high rates of apnea +hypopnea ≥ 1/hour, hypoxemia, hyperscapnia, sleep fragmentation, and indirect evidence of increased airway resistance. With hypopnea episodes being part of the diagnostic criteria - not only the apnea episodes, cases interpreted as “primary snoring” are currently considered “mild OSAS”. Since the hypopnea events are more subtle, the polysomnographic diagnosis of OSAS has been frequent in children, although parents do not report any important obstructive respiratory event during the night.

In pre-surgical assessment, the role of diagnostic polysomnography becomes increasing more important do define borderline cases from the clinical standpoint, especially because the clinical predictors, such as tonsil size, have contradictory results: it is common to have children with severe OSAS and non-obstructive tonsils, and vice-versa.

If, on the one hand polysomnography tests become increasingly relevant, on the other hand it must not be used as the sole tool used to classify disease severity. Children with the same apnea + hypopnea indices may have different clinical involvements, and a child with a higher index may be clinically better than another with a lower index. In general, cases with 1/h ≤HAI ≤ 5/h have mild involvement, and cases of HAI >5/h have moderate to severe involvement, depending on a more global evaluation of the polysomnography and, mainly, the clinical picture presented by the child.

From the treatment standpoint, we have also gone through some rethinking. Adenotonsillectomy, traditionally curative for most of the cases, shows, based on new residual OSAS criteria, very disappointing cure rates of 25–60%. Results also vary according to methodological differences in the definition of residual OSAS; the strictest - IAH ≤ 1/h, was associated to the resolution of only 27% of the cases in a recent multicentric study, with the caveat that approximately 50% of the sample was made up of obese patients. The most mentioned predictors of residual disease are obesity and severe disease at the time of the diagnosis. It is still unclear how much the medical interview during post-surgical evaluation is able to tell cured cases from residual cases. Without doubts, it must include not only questions about the presence of snoring and respiratory pauses during sleep, but also about restless sleep. Cognitive and behavioral improvements may or may not happen.

Both from the diagnostic as well as the treatment standpoint we are seeing an ideal medicine, supported by complementary tests, and a possible medicine, as it is for so many other clinical entities. Having said that, when should one order a polysomnographic test? Such theme must lead to new standardizations. For now, it seems reasonable to order a polysomnographic study before tonsillectomy at least for: children with comorbidities; obese children; when suspecting a child has severe OSAS; and in order to define controversial cases. Polysomnography must be indicated after surgery in cases in which symptoms persist and in children with severe OSAS before the procedure.

Paradoxically, new concepts put us face-to-face with old beliefs: anamneses must be careful in the pre and postoperative evaluations; each child must be assessed in a more holistic way; and, when the complementary test is available, it may not be able to establish clinical severity, and each should be assessed in an individual basis.

And finally, we may be far from a cake recipe or a consensus, but we are, certainly, closer to the truth.

